# Broad-spectrum inhibition of influenza A virus replication by blocking the nuclear export of viral ribonucleoprotein complexes

**DOI:** 10.1128/jvi.01478-25

**Published:** 2025-11-25

**Authors:** Wentao Shen, Jie Xu, Zhaoshan Chen, Yanli Wei, Qian Wang, Xiangkun Wang, Xuegang Zhang, Qiyun Zhu, Shuai Xu

**Affiliations:** 1 State Key Laboratory for Animal Disease Control and Prevention, College of Veterinary Medicine, Lanzhou University, Lanzhou Veterinary Research Institute, Chinese Academy of Agricultural Sciences 111658 , Lanzhou, Gansu, China; University of Kentucky College of Medicine, Lexington, Kentucky, USA

**Keywords:** influenza A viruses, nanobody, nucleoprotein, nuclear export, viral replication

## Abstract

Influenza A virus (IAV) remains a major threat to global health. The emergence of IAV strains resistant to commonly used anti-influenza medicines has made the need for novel broad-spectrum antiviral agents more urgent. The IAV nucleoprotein (NP) is one of the most conserved viral proteins encoded by IAV and is essential for multiple processes in the lifecycle of IAVs. In this study, we screened a high-affinity nanobody, Nb7, which specifically recognizes NP from different subtypes of IAV and broadly inhibits the replication of various IAVs. Mechanistically, Nb7 blocked the nuclear export of viral ribonucleoprotein (vRNP) complexes and the subsequent assembly of progeny virions by specifically binding the nuclear export sequence 1 (NES1) region of the NP. The conserved Q42/E46/K48 residues within the NES1 region of NP were identified as the key sites for the binding of Nb7 and were synergistically responsible for the nuclear export of vRNP. In addition, Nb7 fused with a trans-activating transduction peptide efficiently inhibited the replication of IAV *in vitro* and provided full protection against lethal IAV infection in a mouse model. Taken together, our findings revealed that the NP-specific Nb7 is a promising candidate against IAV infection. This study provides a new understanding of the nuclear export of IAV-vRNP and contributes to the development of broad-spectrum anti-influenza approaches.

**IMPORTANCE:**

Influenza A viruses (IAVs) cause seasonal epidemics and global pandemics, resulting in hundreds of thousands of deaths annually. The identification of conserved epitopes and the development of effective prevention and treatment strategies are crucial for addressing these challenges. The nucleoprotein (NP) protein of IAV is a key component of the viral ribonucleoprotein (vRNP) complex and is highly conserved in various subtypes. Therefore, the development of vaccines or drugs that target conserved viral proteins such as the NP is promising. We screened Nb7, which specifically recognizes NP and broadly inhibits IAV replication through blocking the nuclear export of vRNP. More importantly, the trans-activating transduction peptide-fused Nb7 has promising effects for the prevention and treatment of IAV infection. Our present study provides novel insight for the further development of broad-spectrum vaccines and anti-influenza drugs.

## INTRODUCTION

Influenza A virus (IAV) remains a significant threat to global health, with approximately 1 billion seasonal influenza cases worldwide each year, resulting in 290,000–650,000 deaths (1). Due to persistent antigenic shift and drift, IAVs continually cross the species barrier to transmit and infect mammals, including humans, cows, and sheep, posing a significant threat to public health (2). Identifying new vulnerabilities in the viral life cycle is critical for the development of effective therapeutic strategies. Traditional anti-influenza drugs and vaccines target mainly viral surface proteins. However, surface proteins tend to mutate under immune stress, which significantly reduces the effectiveness of antiviral methods. Therefore, the development of broad-spectrum antiviral agents that target more conserved proteins of IAV is important.

IAV belongs to the *Orthomyxoviridae* family and encodes at least 14 viral proteins, including basic polymerase 2 (PB2), basic polymerase 1 (PB1), acidic polymerase (PA), hemagglutinin (HA), nucleoprotein (NP), neuraminidase (NA), matrix (M) proteins, and nonstructural (NS) proteins (3). The viral ribonucleoprotein (vRNP) complexes formed by polymerase proteins (PB1, PB2, and PA), the NP, and the viral genome vRNA are the basic functional units of viral replication and transcription (4). In the early stage of infection, vRNP enters the nucleus to initiate replication and transcription of the viral genome. In the late stage of infection, the newly assembled vRNPs are exported from the nucleus to form progeny virions (5).

NP, the primary protein that forms the vRNP complex, guarantees the formation of the IAV genome into vRNPs with the correct conformation and structure (6). The vRNP complex needs to form an inverse parallel double helix structure through interactions between NP oligomers. Concurrently, three nuclear localization signals (NLSs), three functional nuclear export signals (NESs), and at least one nuclear accumulation signal have been identified in the NP (7–11). vRNPs rely on these signal sequences of the NP to mediate its transport between the cytoplasm and the nucleus (10, 12–16). Therefore, as one of the most conserved proteins among IAV virions, the NP plays an integral role in the life cycle of IAV, making the NP an ideal target for developing broad-spectrum methods against IAVs.

Nanobodies (Nbs) are variable domain fragments derived from camel heavy-chain antibodies and are characterized by strong physical and chemical stability, as well as low immunogenicity (17). Nbs can be expressed efficiently in a wide range of different expression systems at a low cost (18). Compared with conventional antibodies, Nbs have a longer complementary-determining region (CDR), enabling them to bind to the channel, seam, or hidden epitopes of the target proteins, thereby modulating their functions (19). Therefore, Nbs are valuable for the development of novel antiviral agents (20).

The present study aimed to screen for broad-spectrum Nbs and identify novel targets against IAV infection. We immunized alpaca and obtained Nb7, which broadly inhibited IAV. Nb7 recognizes the NP Q42/E46/K48 sites, which are located in the NES1 region, to block the nuclear export of vRNP. Notably, the Q42/E46/K48 triple mutation abolished the ability to mediate the nuclear export of vRNP. After fusion with the trans-activating transduction (TAT) peptide, Nb7 protected the mice from lethal IAV infection. Together, this study lays the groundwork for the development of novel broad-spectrum anti-influenza strategies.

## RESULTS

### Screening of NP-specific Nbs with broad-spectrum inhibitory effects on IAVs

**Fig 1:**
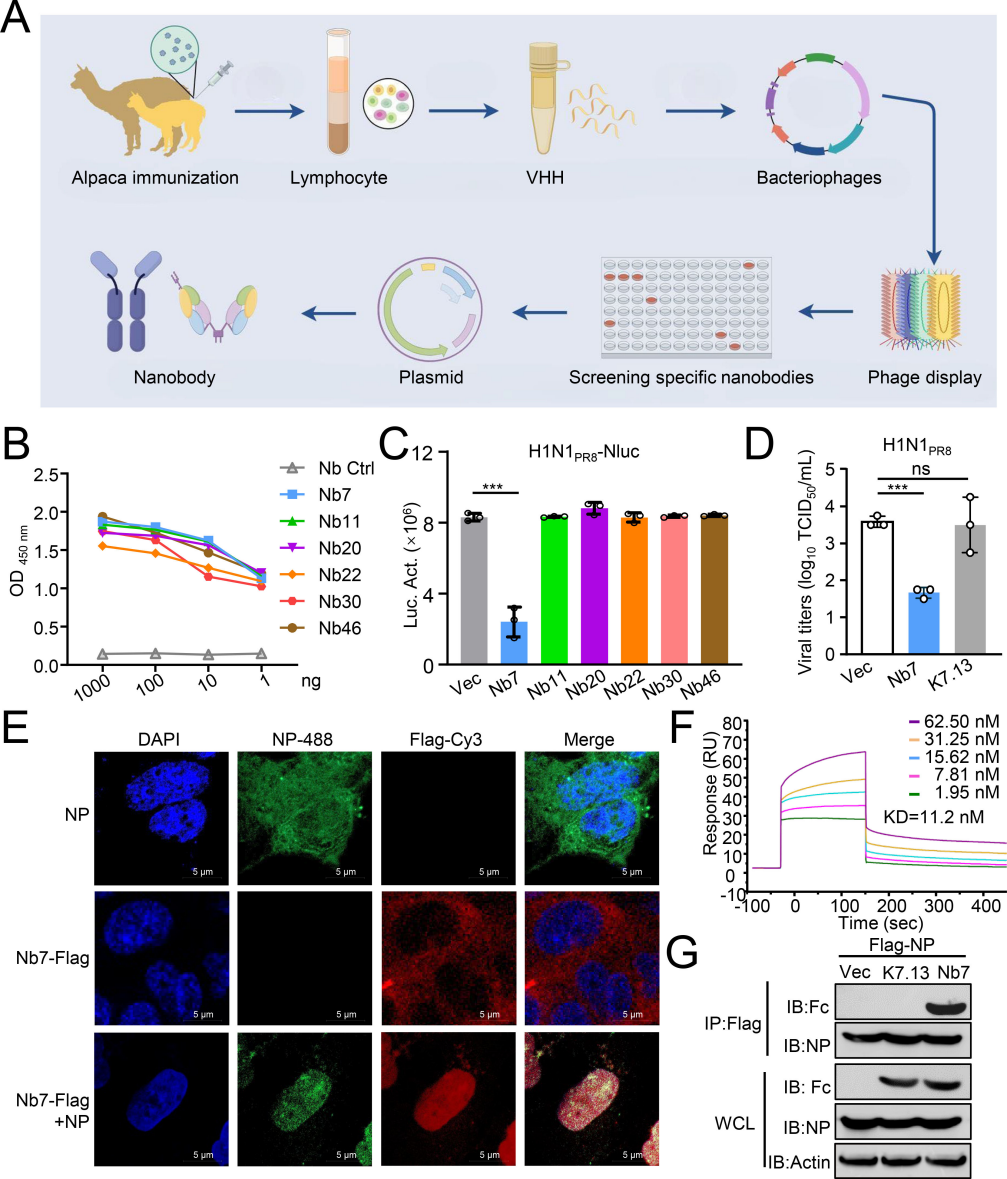
Screening and characterization of NP-specific Nbs. (**A**) Schematic illustration of the alpaca immunization process in alpacas and subsequent Nb screening. (**B**) The binding activity of the indicated Nbs (2 µg/mL) with different doses of NP, as measured by indirect ELISA. (**C**) A549 cells were transfected with the indicated Nbs expression plasmid or empty vector (Vec). After 24 h, the cells were infected with the PR8-Nluc virus for an additional 24 h, and the supernatants were collected to detect luciferase activity by Nano-Glo luciferase assay system (Promega). (**D**) A549 cells were transfected with Nb7-Flag, K7.13-Flag, or Vec for 24 h before infection with the PR8 virus (H1N1, multiplicity of infection [MOI] = 0.01). The supernatants were collected 24 h post-infection for the TCID_50_ assay. (**E**) A549 cells were transfected with Nb7-Flag and/or NP for 24 h before being fixed and stained with anti-Flag (diluted 1:300) and a commercial anti-NP mAb (diluted 1:300). (**F**) Biocore T200 detected the affinity of Nb7-Fc with PR8-NP. (**G**) HEK293T cells were transfected with PR8-NP, along with Vec, K7.13-Fc, or Nb7-Fc, for 24 h. The cell lysates were coimmunoprecipitated with an anti-Flag antibody. The data shown represent three independent experiments (*n* = 3); the bars represent the means ± SDs.

To identify NP-specific Nbs with broad-spectrum inhibition of IAV replication, recombinant NP protein derived from the H1N1 IAV (PR8 strain) was expressed and purified from HEK293T cells and was used as an antigen for immunizing alpacas and screening Nbs (Fig. 1A). After three rounds of screening, 48 clones were selected for indirect enzyme-linked immunosorbent assay (ELISA) to detect the reaction with the NP protein purified from *E. coli* cells (Fig. S1A). The phage-ELISA results indicated that Nb7, Nb11, Nb20, Nb22, Nb30, and Nb46 were highly reactive with the NP, with an absorbance value greater than 2. Amino acid sequence analysis revealed that the CDR3 region of the six Nb strains is composed of different amino acid residues (Fig. S1B). By fusion with human Fc fragments, six recombinant Nbs, that is, Nb7-Fc, Nb11-Fc, Nb20-Fc, Nb22-Fc, Nb30-Fc, and Nb46-Fc, were successfully expressed and purified from HEK293F suspension cells (Fig. S1C). Indirect ELISA suggested that all six recombinant Nbs strongly bind to NP (Fig. 1B).

Using an IAV reporter virus (PR8-Nluc), we found that transiently expressed Nb7 significantly inhibited IAV replication, whereas other Nbs did not (Fig. 1C; Fig. S1D). Moreover, transiently expressed Nb7 significantly inhibited the replication of the wild-type H1N1-PR8 virus in cells, but the negative control Nb (K7.13), which specifically recognizes the nCoV S protein, did not affect the replication of H1N1-PR8 (Fig. 1D; Fig. S1E) (21). The indirect immunofluorescence assay (IFA) indicated that Nb7 colocalized with PR8-NP in the nucleus of cells transfected with plasmids encoding NP and Nb7 genes (Fig. 1E). Using surface plasmon resonance method to detect the affinity between the NP and Nb7, the equilibrium dissociation constant (KD) between the NP and Nb7 was determined to be 11.2 nM (Fig. 1F), which indicated that Nb7 has a high affinity with NP. In addition, purified Nb7 immunoprecipitated with NP protein in transfected cells and the purified GST-NP, but K7.13 Nb did not (Fig. 1G; Fig. S1F).

**Fig 2:**
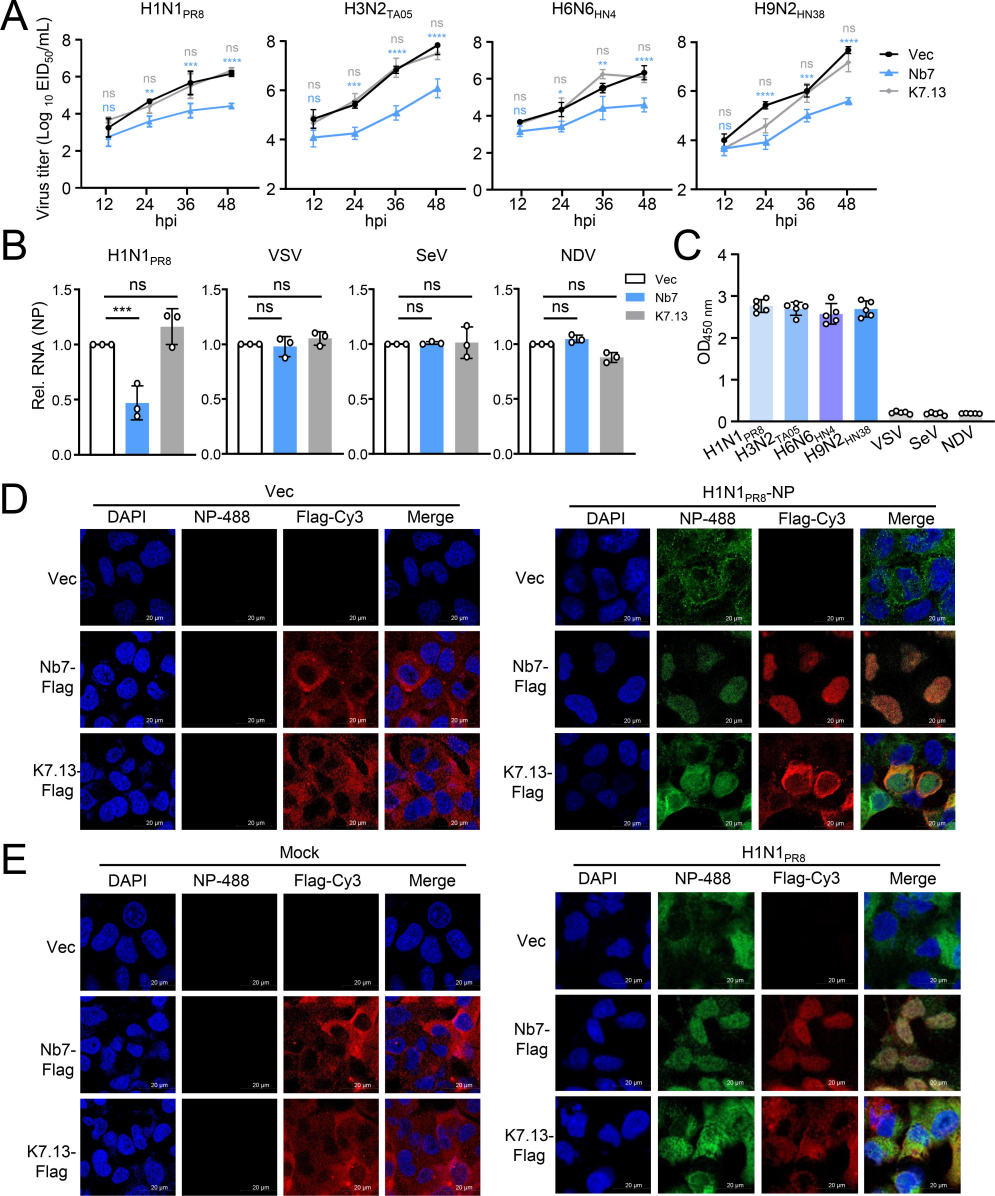
The Nb7 antibody specifically recognizes IAV NP and inhibits IAV replication. (**A**) A549 cells were transfected with Nb7-Flag, K7.13-Flag, or Vec. After 24 h, the cells were infected with the PR8 (H1N1), TA05 (H3N2), HN4 (H6N6), or HN38 (H9N2) viruses (MOI = 0.1). At the indicated times post-infection, the supernatant containing the viral particles was collected for the EID_50_ assay. The significant difference between Nb7 and Vec is labeled in blue; the significant difference between K7.13 and Vec is labeled in gray. (**B**) A549 cells were transfected with Nb7-Flag, K7.13-Flag, or Vec. After 24 h, the cells were infected with PR8 (H1N1), VSV, SeV, or NDV for an additional 24 h. Total RNA was then extracted for qPCR to detect the expression of viral RNA. (**C**) Indirect ELISA was used to detect the ability of Nb7 to recognize different viruses. The purified viral particles (2 µg/mL) of different viruses were coated on 96-well microtiter plates, and Nb7-Fc (2 µg/mL) was then added. (**D**) A549 cells were transfected with Nb7-Flag, K7.13-Flag, or Vec, along with Vec or H1N1_PR8_-NP. After 18 h, the cells were fixed and stained with anti-Flag (diluted 1:300) and a commercial anti-NP mAb (diluted 1:300). (**E**) A549 cells were transfected with Nb7-Flag, K7.13-Flag, or Vec. After 24 h, the cells were infected with PR8 (H1N1) virus (MOI = 0.1) or Mock for 24 h and stained with anti-Flag (diluted 1:300) and a commercial anti-NP mAb (diluted 1:300). The data shown represent three independent experiments (*n* = 3); the bars represent the means ± SDs.

We subsequently verified the effect of Nb7 on the replication of various IAVs. The replication kinetics curve revealed that transiently expressed Nb7 significantly reduced the replication of H1N1, H3N2, H6N6, and H9N2 viruses (Fig. 2A). Moreover, compared with K7.13 and the Vec control, the expression of Nb7 significantly decreased the expression levels of viral RNA and protein in H1N1, H3N2, H6N6, and H9N2 IAV subtypes (Fig. S2A and B). Moreover, transiently expressed Nb7 did not affect the replication of other viruses, such as vesicular stomatitis virus (VSV), Sendai virus (SeV), or Newcastle disease virus (NDV) (Fig. 2B). The ELISA results confirmed that purified Nb7 specifically recognized H1N1, H3N2, H6N6, and H9N2 IAVs, but not VSV, SeV, or NDV (Fig. 2C). Additionally, both Nb7 and K7.13 were mainly distributed in the cytoplasm of transfected cells. However, the distribution of Nb7 was rearranged and colocalized with NP in the nucleus following transfection with NP or infection with H1N1, H3N2, H6N6, and H9N2 viruses (Fig. 2D and E; Fig. S2C and D).

These results indicated that Nb7 specifically binds to NP with high affinity and broadly inhibits the replication of different subtypes of IAV in cells.

### Nb7 inhibits viral replication by blocking the nuclear export of vRNP

**Fig 3:**
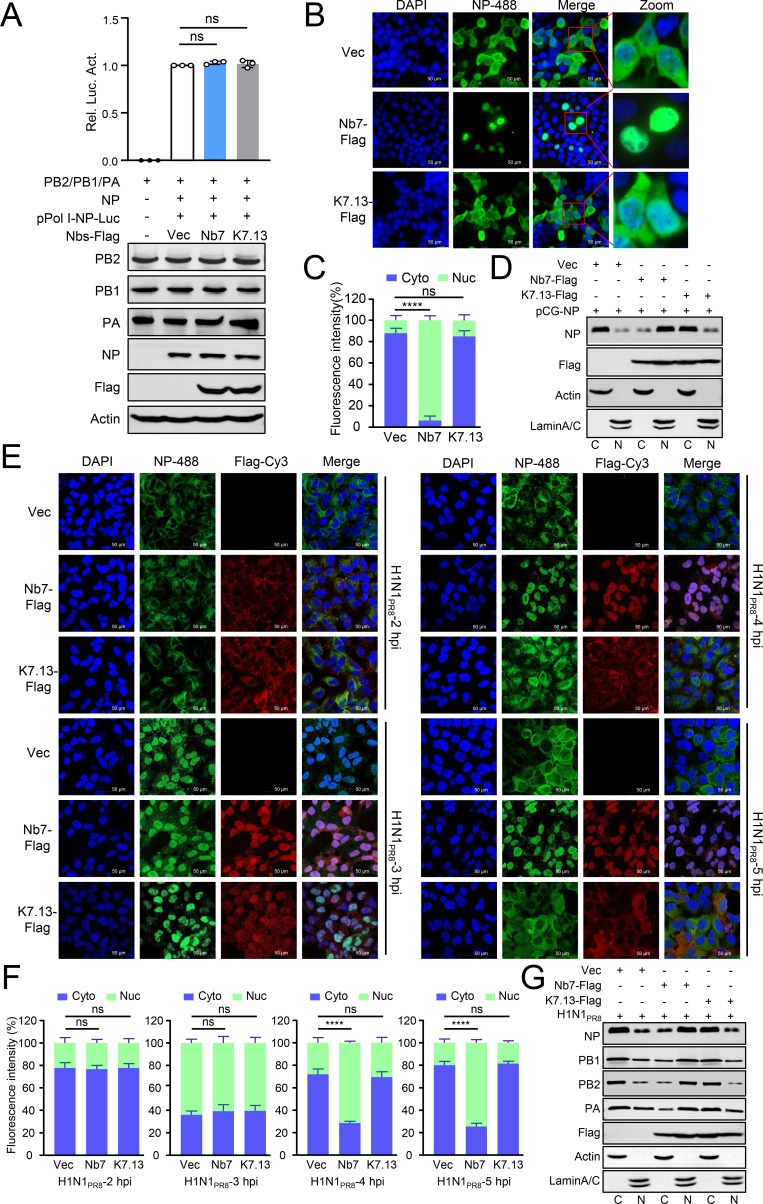
Nb7 inhibits IAV replication by blocking the nuclear export of vRNP. (**A**) HEK293T cells were transfected with pCAGGS (pCG) constructs expressing viral PB2, PB1, PA, and NP proteins from the PR8 virus, the pPol I-Luc construct, and an internal control pRL-TK (Promega), along with Vec, Nb7-Flag, or K7.13-Flag plasmids, for 24 h, and the cell lysates were analyzed via a luciferase assay. The data shown represent the ratio of luciferase activity normalized to that of the control group. (**B**) A549 cells were transfected with Nb7-Flag, K7.13-Flag, or Vec along with pCG-PR8-NP. After 24 h, the cells were fixed and stained with a commercial anti-NP mAb (diluted 1:300). (**C**) The subcellular distribution of the NP in (**B**) was calculated via ImageJ. At least 30 cells from each group were counted. (**D**) A549 cells were transfected with Nb7-Flag, K7.13-Flag, or Vec along with PR8-NP. After 24 h, the nuclear and cytoplasmic fractions of the cells were separated for Western blotting analysis. (**E**) A549 cells were transfected with Nb7-Flag, K7.13-Flag, or Vec. After 24 h, the cells were infected with the PR8 (H1N1) virus at a multiplicity of infection (MOI) of 10. At 2, 3, 4, and 5 h post-infection, the cells were fixed and stained with anti-Flag (diluted 1:300) and a commercial anti-NP mAb (diluted 1:300). (**F**) ImageJ was used to calculate the subcellular distribution of the NP in (**E**). At least 30 cells from each group were counted. (**G**) A549 cells were transfected with Nb7-Flag, K7.13-Flag, or Vec. After 24 h, the cells were infected with the PR8 (H1N1) virus at an MOI of 10. At 5 h post-infection, the nuclear and cytoplasmic fractions of the cells were separated for Western blotting. The data shown represent three independent experiments (*n* = 3); the bars represent the means ± SDs.

Next, we investigated the mechanism by which Nb7 inhibits IAV replication. Previous studies have shown that the NP encapsulates viral RNAs and associates with three polymerase proteins to form the vRNP complex, which is responsible for the transcription and replication of viral genomes (5, 22, 23). The results of the polymerase activity assay revealed that the expression of Nb7 does not affect viral polymerase activity (Fig. 3A). Furthermore, co-immunoprecipitation (co-IP) and cross-linking RNA immunoprecipitation assays revealed that the expression of Nb7 does not inhibit the oligomerization of NP or the interaction of NP with viral RNAs (Fig. S3A and B). However, the distribution of the NP protein revealed that there are more NP signals in the nuclei and less in the cytoplasm of cells expressing Nb7 than in those expressing K7.13 or the Vec control (Fig. 3B and C). Consistently, the cell fraction isolation assay indicated that more NP proteins were detected in the nuclear fraction from Nb7-expressing cells than in that from K7.13-expressing cells (Fig. 3D).

NP is the main component protein of the vRNP complex and is responsible for mediating the nuclear import and export of vRNP (5). The distribution of NP proteins partially represents the localization of vRNPs within cells, especially during the early stages of infection. Two hours after viral infection, the vRNPs were located in the cytoplasm and did not differ in either Nb7- or K713-expressing cells, indicating that Nb7 did not affect the attachment or internalization of IAV (Fig. 3E). At 3 h after infection, most of the NP proteins were located in the nucleus, and no significant differences were detected among the Vec-, Nb7-, or K7.13-expressing cells, indicating that Nb7 did not affect the nuclear import of vRNP. At 4 and 5 h after infection, the NP proteins were mostly distributed in the cytoplasm and tended to aggregate at the cell membrane in Vec- and K7.13-expressing cells. In contrast, most NP proteins were still located in the nucleus of the Nb7-expressing cells (Fig. 3E and F). Similar results were obtained from the cell fraction isolation assay, which revealed that Nb7 promoted the accumulation of NP and polymerase proteins in the nuclei of virus-infected cells (Fig. 3G). These results demonstrated that Nb7 blocks the nuclear export of the vRNP complex.

### Nb7 specifically recognizes conserved residues in the NES1 region of the NP

**Fig 4:**
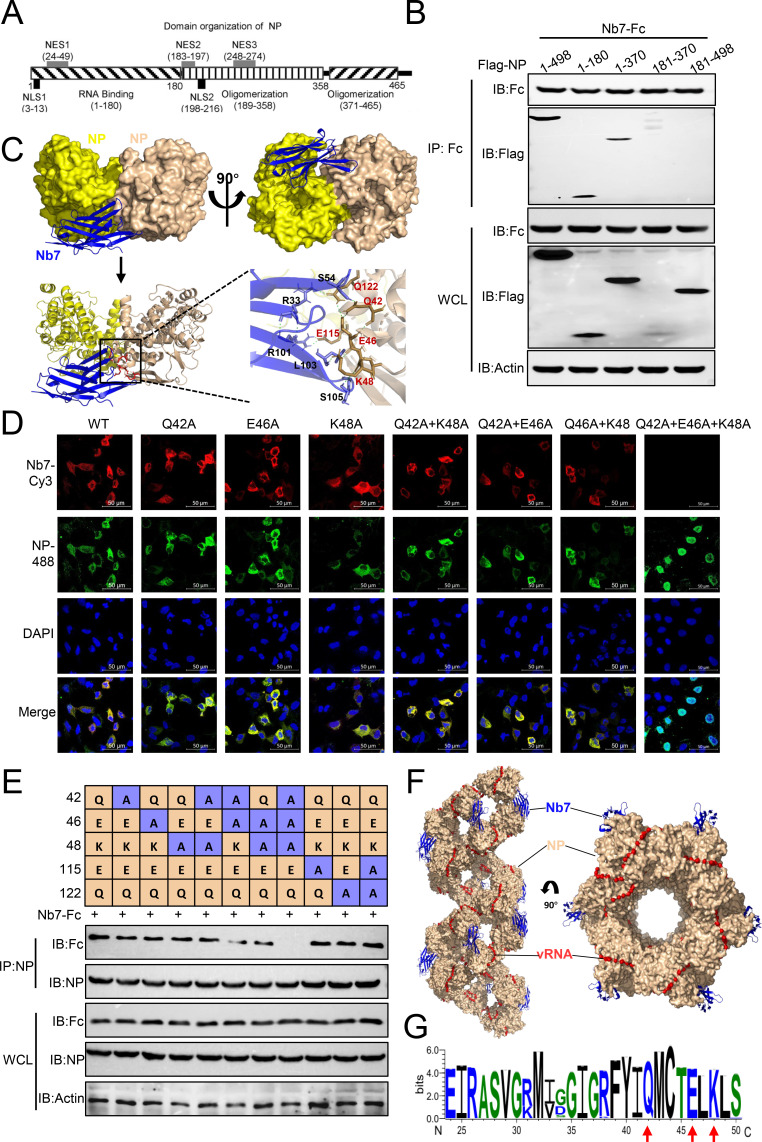
The binding site of Nb7 is located in the NES1 region of the NP. (**A**) Schematic representation of the organization of the NP domain. (**B**) HEK293T cells were transfected with Nb7-Fc along with NP or NP truncations. After 24 h, the cell lysates were co-IP with an anti-human Fc mAb. (**C**) Structure prediction and molecular docking were performed via the AlphaFold 3 online tool (NP oligomer: buff; Nb7: blue). The stick represents the interaction residue of Nb7 with the NP dimer. The dashed green lines represent hydrogen bonds. The predicted antigen-antibody interchain-predicted alignment error in the contact region was generally lower than 5 Å, and the pLDDTs of the key interface residues were all greater than 85. (**D**) A549 cells were transfected with NP or the indicated NP mutants. After 18 h, the cells were fixed and stained with a commercial anti-NP mAb (diluted 1:300) and Nb7-Fc (2 µg/mL). (**E**) HEK293T cells were transfected with NP-Fc along with NP or the indicated NP mutants. After 24 h, the cell lysates were co-IP with a commercial anti-NP mAb. (**F**) Superposition of the Nb7 structure on the vRNP model (PDB ID: 2YMN) from the work of PyMOL (Nb7: blue; NP oligomer: buff). (**G**) NP amino acid sequences of all subtypes of IAVs. NP sequences were downloaded from GenBank via the “collapse identical sequences” option. The logo was generated via the WebLogo3 online tool (weblogo.threeplusone.com). The data shown represent three independent experiments (*n* = 3); the bars represent the means ± SDs.

To further elucidate the mechanism by which Nb7 inhibits the replication of IAV, we next identified the binding sites of Nb7 on the NP protein. We constructed multiple NP truncations based on the domain organization of the NP (24). Nb7 recognizes the RNA-binding domain (aa 1–180) of the NP (Fig. 4A and B). The complex structure of PR8-NP and Nb7 was predicted via AlphaFold 3. The prediction results for the NP protein were consistent with the known structural model, indicating that the prediction of this binding interface is highly confident. The predictive model revealed that Nb7 mainly binds to a protruding area on the antigen surface through its CDRs. The key interaction interfaces include R101, L103, and S105 of CDR3, as well as R33 and S54 of CDR2. L103 forms crucial hydrogen bonds with E46 and K48 of the NP, whereas R33, S54, R101, and S105 of Nb7 form salt bridges and hydrogen bond networks with Q42, E115, and Q122 of the NP (Fig. 4C). Notably, residues Q42, E46, K48, E115, and Q122 are located within the RNA-binding domain of the NP, which is consistent with the former results shown in Fig. 4B.

To identify the key residues of NP recognized by Nb7, a series of single and multiple mutant plasmids was constructed. Using purified Nb7-Fc as the first antibody, the IFA results confirmed that the NP-Q42A/E46A/K48A mutant was not recognized by Nb7. In contrast, the single or double mutants were still recognized by Nb7 (Fig. 4D). In addition, triple-mutant NP (Q42A/E46A/K48A) accumulated predominantly in the nucleus. In contrast, the single or double mutants were distributed in both the nucleus and the cytoplasm (Fig. 4D). Moreover, the co-IP results demonstrated that Nb7 completely lost the ability to recognize NP when the Q42, E46, and K48 residues were simultaneously mutated (Fig. 4E). Considering that Q42, E46, and K48 are located in the NES1 region of the NP, we constructed NES1 and NLS1 truncations and detected that the NES1 truncation was located mainly in the nucleus and was not recognized by Nb7 (Fig. S4A and B). Furthermore, we superimposed the vRNP structure and Nb7 (25). As shown in Fig. 4F, multiple binding sites exist between Nb7 and vRNP, all of which are exposed on the outer surface of the rod-like vRNP structure, allowing various Nb7 molecules to bind to the NP within the vRNP complex. The alignment of 76,616 NP sequences of all the IAVs from GenBank revealed that the Q42, E46, and K48 residues are highly conserved in the NP among all the IAVs (Fig. 4G).

The data above demonstrated that Nb7 recognizes the Q42, E46, and K48 residues located in the conserved NES1 region of the NP and that the triple mutation (Q42A/E46A/K48A) increases the nuclear retention of the NP.

### TAT-Nb7 enables penetration of the cell membrane and inhibits viral replication

**Fig 5:**
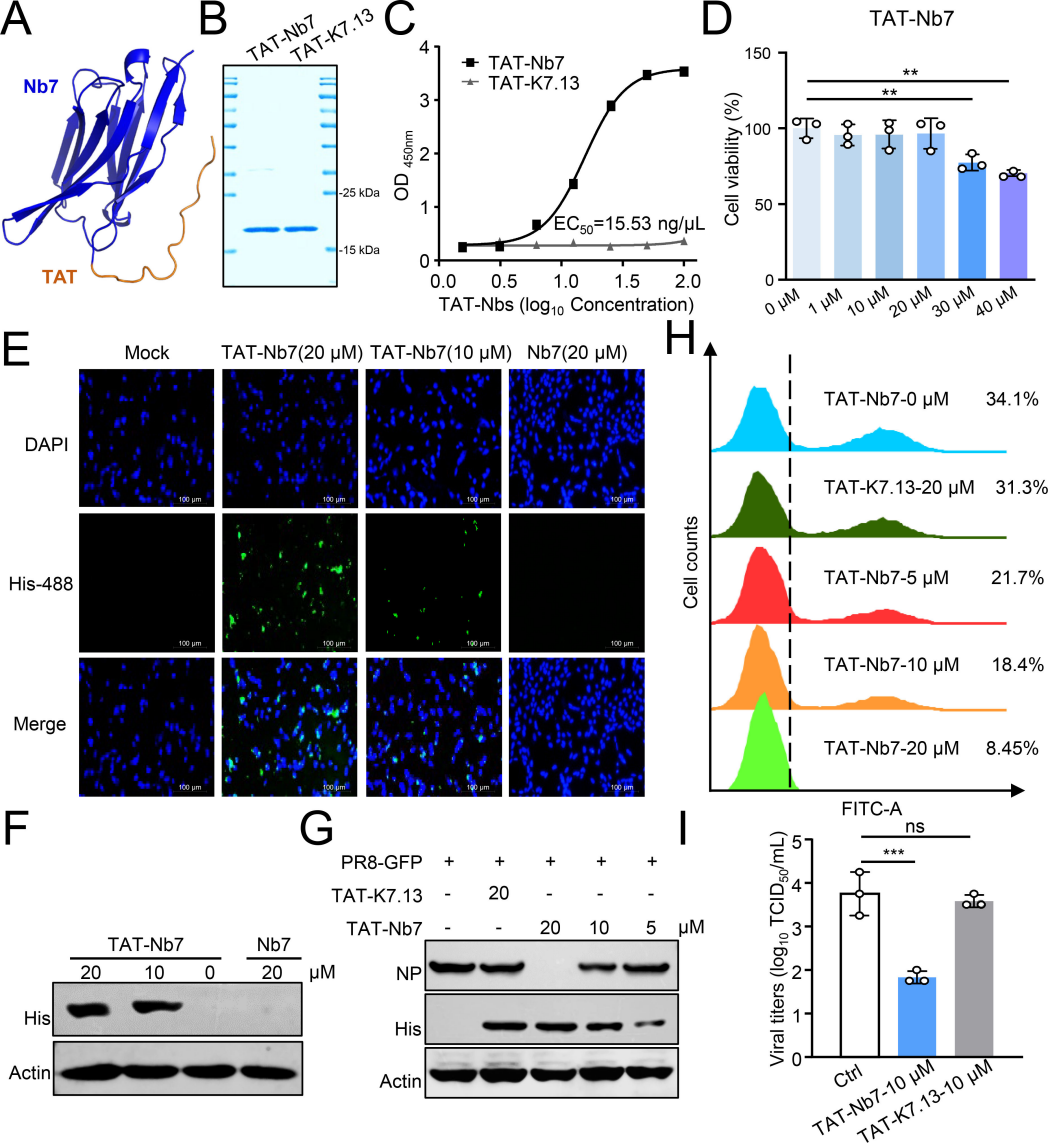
TAT-Nb7 penetrates the cell membrane and inhibits IAV replication. (**A**) A structural model of TAT-Nb7 was constructed via the AlphaFold 3 online tool (Nb7: blue; TAT: orange). (**B**) The expression of purified TAT-Nbs was analyzed by SDS-PAGE. (**C**) ELISA-binding curves showing the interaction of TAT-Nbs with PR8-NP (2 µg/mL). (**D**) Different doses of TAT-Nb7 were added to the culture media of A549 cells. After 24 h, cell viability was determined via a CCK8 assay. (**E**) Different concentrations of TAT-Nb7 or Nb7 were added to the cell supernatant of A549 cells. After 6 h, the cells were fixed and stained with an anti-His antibody (diluted 1:300). (**F**) Different concentrations of TAT-Nb7 or Nb7 were added to the cell supernatant of A549 cells. After 6 h, the cell lysates were subjected to Western blotting analysis. (**G and H**) Different concentrations of TAT-Nb7 or TAT-K7.13 were added to the cell supernatant of A549 cells. After 6 h, the cells were infected with the PR8-GFP virus (multiplicity of infection [MOI] = 0.1). At 24 h post-infection, the cells were collected for Western blotting (**G**) and flow cytometry (**H**). (**I**) 10 µM of TAT-Nb7 or TAT-K7.13 was added to the supernatant of A549 cells. After 6 h, the cells were infected with the PR8-GFP virus (MOI = 0.1). At 24 h post-infection, the supernatants were collected for the TCID_50_ assay. The data shown represent three independent experiments (*n* = 3); the bars represent the means ± SDs.

As NP is an intracellular viral protein, we used a TAT peptide derived from human immunodeficiency virus type 1 (HIV-1) to deliver Nb7 into cultured cells and tissues through the fusion of the TAT peptide to the N-terminus of Nb7 (Fig. 5A) (26, 27). SDS-PAGE analysis confirmed that TAT-Nb7 and TAT-K7.13 were successfully expressed and purified (Fig. 5B). Indirect ELISA results revealed that TAT-Nb7 maintained high reactivity with the NP (Fig. 5C). The results of the CCK8 assay indicated that TAT-Nb7 did not affect cell viability at concentrations less than or equal to 20 µM (Fig. 5D). As shown in Fig. 5E and F, TAT-Nb7 was efficiently taken up by cells in a dose-dependent manner, but Nb7 was not. Furthermore, TAT-Nb7 inhibited the expression of viral proteins and the replication of the PR8-GFP virus in a dose-dependent manner (Fig. 5G and H). Similarly, TAT-Nb7 significantly inhibited the replication of the wild-type PR8 virus in cells (Fig. 5I). These data suggest that the TAT peptide efficiently delivers Nb7 into cells and inhibits the replication of IAV.

### TAT-Nb7 treatment protects mice against lethal virus challenge

**Fig 6:**
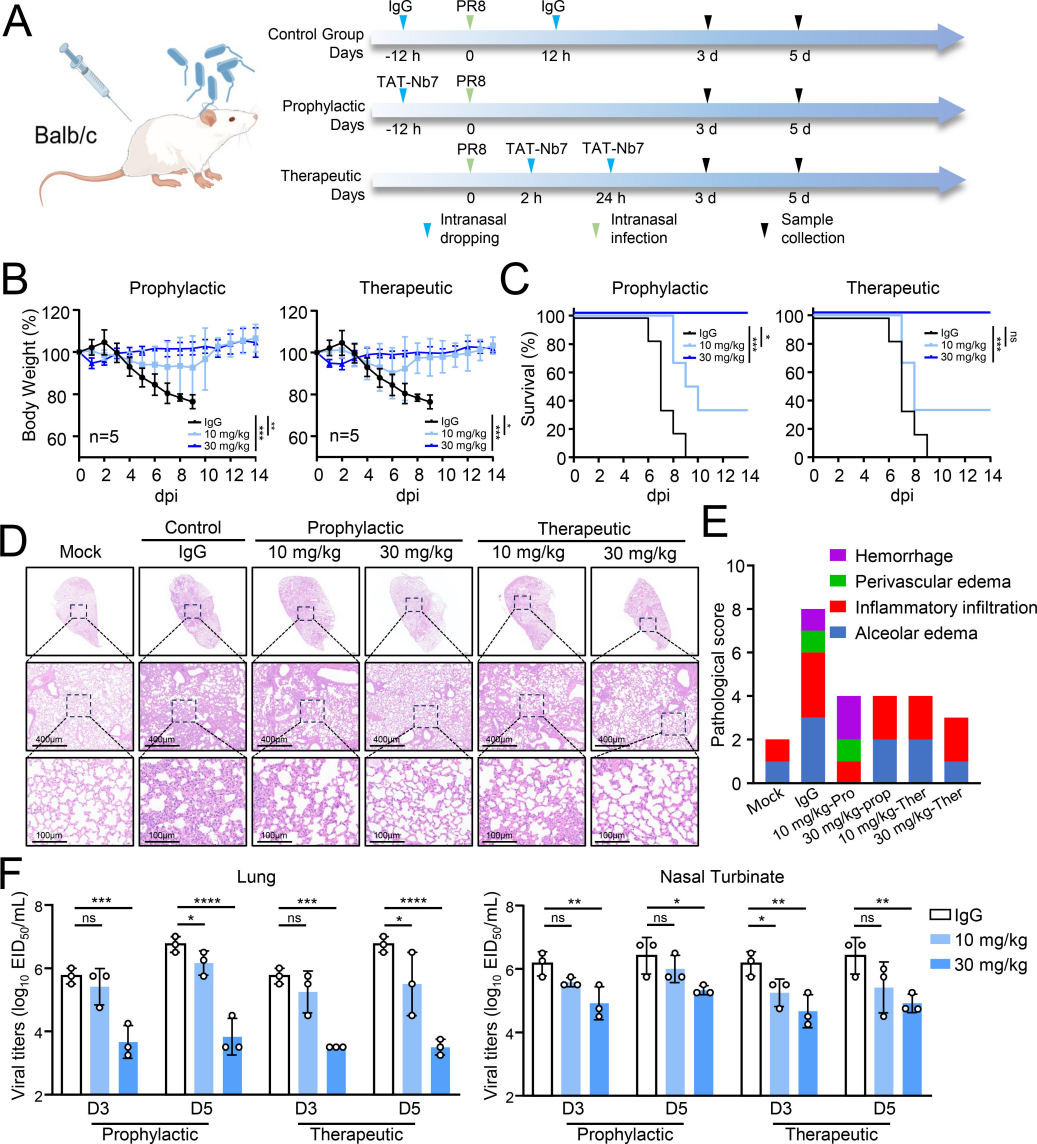
Nb7 treatment protects mice against IAV challenge. (**A**) Schematic representation of the experimental design for the mouse study. (**B and C**) Body weight and survival changes were monitored for 14 days after virus challenge. (**D**) Representative histopathological analysis of lungs from mice on days 3 and 5 after PR8 (H1N1) infection, as outlined in A. Scale bars, 1,000 µm (top), 400 µm (middle), and 100 µm (bottom). (**E**) Scores of pathological sections from the mice in (**D**). (**F**) TAT-Nb7 and IgG-treated mice intranasally infected with PR8 (10^4^ EID_50_) were euthanized on days 3 and 5 post-inoculation, and the lungs and nasal turbinates were collected for the EID_50_ assay, as described in A. The data shown represent three independent experiments (*n* = 3); the bars represent the means ± SDs.

We further evaluated whether TAT-Nb7 exhibits protective efficacy against IAV infection *in vivo*. For prophylactic assessment, mice were administered either a low (10 mg/kg) or high (30 mg/kg) dose of TAT-Nb7 or IgG intranasally at 12 h prior to infection, followed by infection with 10^4^ EID_50_ of the PR8 virus. For therapeutic assessment, mice were administered either a low (10 mg/kg) or high (30 mg/kg) dose of TAT-Nb7 intranasally at 2 and 24 h post-infection (Fig. 6A).

For the prophylactic group of mice challenged with the PR8 virus, TAT-Nb7 provided 100% protection at a dose of 30 mg/kg and 40% protection at a dose of 10 mg/kg (Fig. 6B and C). As shown in Fig. 6D, virus infection resulted in severe damage to the lung, characterized by diffuse alveolar damage, including thickening of the alveolar septa, marked epithelial hyperplasia in the bronchi/bronchioles, and extensive immune infiltration in the alveoli, bronchi, and vessels. The overall pathology scores were significantly lower in the mice pretreated with TAT-Nb7 (Fig. 6E). Following prophylactic treatment with TAT-Nb7, the viral load in the mouse tissues decreased significantly (Fig. 6F).

Next, we evaluated the therapeutic efficacy of TAT-Nb7 against IAV. As shown in Fig. 6B and C, TAT-Nb7 at a dose of 30 mg/kg provided complete protection for mice challenged with a lethal infection. The lung lesions of the mice treated with TAT-Nb7 were significantly alleviated (Fig. 6D and E). Moreover, following treatment with TAT-Nb7 at 30 mg/kg, the viral load in tissues was lower than that in mice treated with IgG (Fig. 6F).

Overall, the data indicated that TAT-Nb7 reduced the replication and pathogenicity of IAV, displaying promising protective efficacy against IAV infection.

## DISCUSSION

In the present study, we identified and characterized the Nb7 Nb, which specifically recognizes IAV NP. Nb7 inhibits IAV replication by blocking the nuclear export of the vRNP complex. Importantly, we found that the synergistic effects of the NP Q42, E46, and K48 residues are essential for the nuclear export of vRNP. Nb7 fused to the transmembrane peptide TAT displayed promising protection against lethal IAV challenge. These findings provide novel insights for the development of antiviral approaches.

IAVs pose a persistent threat to public health and are characterized by the capacity to undergo antigenic drift and shift rapidly. IAVs constantly cross the interspecies barrier and infect various hosts, as seen in the recent H5N1 infection in cows and sheep in the United States (28). The development of more effective and broad-spectrum strategies to cope with the frequent mutations of IAVs is urgently needed. Compared with surface proteins, internal proteins of IAV are generally more conserved, facilitating the development of broad-spectrum antiviral strategies (29). NP is one of the most conserved IAV proteins, plays a crucial role in the assembly of vRNPs, and facilitates nucleus-cytoplasm shuttling. In the present study, we screened an NP-specific Nb7 with high affinity and broad-spectrum inhibition of IAV replication.

The surface proteins of IAV, that is, HA, NA, and M2, have long been the primary targets for the development of antiviral strategies (30, 31). However, recent investigations revealed that small molecules and other agents targeting NP, PB1, M1, PB2, and other internal viral proteins exert promising antiviral effects (32–35). More recently, Liu et al. reported that naproxen, which targets NP at conserved residues, blocked the interaction between CRM1 and NP and suppressed IAV replication *in vivo* and *in vitro* (36). Ashour et al. isolated several NP-targeting Nbs that exerted anti-IAV activity by blocking the nuclear import of vRNPs and viral transcription and replication in the nucleus (37). The high-resolution crystal structure revealed that Nbs inhibited the nuclear import of vRNPs by occupying the non-conserved surface of the NPs and ingeniously mimicked the antiviral mechanism of the host Mx protein (37–39). Our data revealed that Nb7 inhibits the nuclear export of vRNP, thereby suppressing the assembly of progeny virions and the replication of IAVs. The conserved key sites targeted by Nb7 were identified as crucial for the nuclear export of vRNP. In conclusion, conserved internal viral proteins are promising targets for the development of novel antiviral strategies.

While the conservation of the NP across all IAVs has positioned it as an attractive target, therapeutic exploitation against the NP has been hindered by a limited understanding of its functional epitopes. Our work demonstrated that Nb7 potently inhibits vRNP nuclear export by binding to the NES1 domain, a mechanism distinct from that of existing NP-targeting Nbs, such as VHH1 (39). The NES1 domain of the NP has multiple potential nuclear export motifs (LxxLxL or LxxxLxL), which mediate the nuclear export of nuclear proteins (10, 40). In this study, we determined that the key binding sites of Nb7 are located between the hydrophobic amino acids of the nuclear export motif (FYIQMCTELKL) within the NES1 region of the NP (Fig. 4). Superposition of NP7 on EM-based vRNP models revealed that NP7 could mask the nuclear export motifs on NP molecules (Fig. 4). However, the flexibility and heterogeneity of vRNP complexes pose significant challenges to proving this argument. High-resolution structures of the vRNP complex with interacting proteins may still be needed for confirmation. Importantly, we revealed the synergistic role of the conserved residues Q42, E46, and K48 within NES1. When Q42/E46/K48 is co-mutated, the nuclear export of NP is blocked, as indicated by the accumulation of NP in the nucleus. Notably, the Q42/E46/K48A triple mutation is lethal for IAV. These findings highlighted the synergistic role of the Q42/E46/K48 sites in the function of NP and the life cycle of IAV.

The development of antibody drugs targeting intracellular proteins faces challenges in crossing cellular or capsular barriers to directly bind to target antigens. Currently, lipid nanoparticle (LNP) delivery methods represent promising alternatives for drug delivery. The use of a lung-targeting delivery system to administer mRNAs encoding broadly neutralizing antibodies enables the direct intracellular expression of antibodies against virus infection (41). A major limitation of LNP-mRNA-expressing antibodies in infected cells is the suppression of host protein synthesis, which impairs antibody expression during the late stages of infection. Here, we employed the HIV TAT peptide, derived from successes in HIV therapeutics, to deliver Nb7 into cells. Our results demonstrated that TAT-Nb7 fully protected mice against lethal IAV replication (Fig. 6). The *in vivo* efficacy of TAT-Nb7 suggests that cell-penetrating peptide fusions could overcome the pharmacokinetic barriers that have hindered conventional antibody therapies targeting intracellular proteins such as NP.

Despite the limitations of our present study, such as the lack of definitive structural determination of the Nb7-NP complex via cryo-EM, which may provide more detailed insights into the epitope of Nb, we successfully mapped the binding area of Nb7 as a conserved NES1 domain. In the future, we will attempt to modify Nb7 to improve its affinity and inhibitory effect on IAV replication. Better delivery methods can be employed to assess the *in vivo* applicability of Nb. In conclusion, this study demonstrates that the NP-specific Nb7 is a promising candidate for combating IAV infection. The conserved sites of the NP NES1 region we identified may contribute to the development of a broad-spectrum vaccine and provide insights into novel antiviral approaches.

## MATERIALS AND METHODS

### Biosafety and ethical statements

The details of the facility and the biosafety and biosecurity measures used have been previously reported (42).

### Cells, viruses, and plasmids

HEK293T and MDCK cells (ATCC) were maintained in Dulbecco’s Modified Eagle’s Medium supplemented with 10% fetal bovine serum (FBS; Life Technologies), 100 U/mL penicillin, and 100 µg/mL streptomycin (Life Technologies). A549 cells (ATCC) were grown in Kaighn’s modified Ham F-12 nutrient mixture medium supplemented with 10% FBS, 100 U/mL penicillin, and 100 µg/mL streptomycin. HEK293F cells were maintained in Expi Expression Medium (Gibco, 12338018). All the cells were cultured and maintained at 37°C with 5% CO_2_.

The H1N1 IAV (A/Puerto Rico/8/1934, PR8) was stored in our laboratory. H3N2 IAV (A/Swine/Shandong/TA05/2021) was isolated from Shandong Province, China, in 2021. The H6N6 virus (A/duck/Hunan/4/2018, HN4) was isolated in 2018 from a duck in Henan Province, China. The H9N2 virus (A/chicken/Hunan/38/2018, HN38) was isolated in 2018 from Gansu Province, China. The NDV (MG7 strain) was generated and stored in our laboratory (43). SeV was kindly provided by Hongkui Deng (Peking University, China). As previously described, a recombinant PR8 reporter virus expressing Nanoluciferase (PR8-Nluc) was generated (44). The PR8-GFP virus construction is described in this study (45). Virus stocks were propagated in specific-pathogen-free (SPF) chicken eggs and stored at −70°C until use.

The genes encoding NP/PB1/PB2/PA were constructed via standard molecular biology techniques. The expression of the viral RNA-like firefly luciferase gene under the control of the human RNA polymerase I promoter, via the pPol I-Luc plasmid, has been previously reported (46). All plasmid DNAs were purified from bacteria via a plasmid extraction kit (Omega).

### Reagents and antibodies

The antibodies used in this study were as follows: HRP-conjugated anti-HA (12013819001), anti-Myc (11814150001), and anti-GFP (11814460001) antibodies (Roche); rabbit anti-PB2 (GTX125926), anti-PB1 (GTX125923), and anti-PA (GTX118991) polyclonal antibodies (Genetex); HRP-conjugated anti-human IgG-Fc (SSA001) and anti-NP (11675-MM03T) antibodies (Sino Biological); anti-β actin (TA-09), anti-His tag (TA-02), and HRP-conjugated goat anti-rabbit IgG (ZB-2301) (Zsbio); HRP-conjugated goat anti-mouse secondary antibodies (ab102448); and a Cy3-conjugated anti-Flag antibody (A8592, Sigma).

The following reagents were used in this study: Freund’s adjuvant was purchased from Sigma (Germany); anti-Flag agarose affinity beads (A2220), protease inhibitor cocktail (4693116001), and protein A/G agarose affinity beads (P6486/E3403) (Merck); DAPI (C1002), NP-40 (ST366), 4% paraformaldehyde fix solution (P0099), RNase A (ST578), normal rabbit IgG (A7016), and cell counting kit-8 (CCK8, C0037); Tween-80 (HY-Y1891) (MedChem-Express); and the Expi-Fectamine 293 Transfection Kit (Gibco, A14525). The jetPRIME transfection reagent was obtained from Polyplus (USA). SYBR Green I Master Mix was purchased from Roche (Germany).

### Alpaca immunization and library construction

In consistent with our previous study (47), 1-year-old male alpacas were selected for immunization with recombinant NP purified from HEK293T cells. Five consecutive immunizations were performed every 2 weeks. For the first immunization, 1 mg of purified recombinant NP (1 mg/mL) was emulsified with an equal amount of Freund’s complete adjuvant to immunize alpacas. In the second to fifth immunizations, an equal amount of Freund’s incomplete adjuvant was mixed with purified recombinant NP. After the final immunization, sera were collected from the immunized alpacas and used to test the titer of anti-NP antibodies via indirect ELISA, with recombinant NP (2 µg/mL) purified from *E. coli* cells serving as the coating antigen. Then, peripheral blood lymphocytes were isolated, and total RNA was extracted to synthesize cDNA. The VHH gene fragments were subsequently cloned and inserted into pComb3XSS via nested PCR. The recombinant phage was converted into *E. coli* TG1 cells. The cells were incubated overnight at 37°C on a Luria-Bertani (LB) plate containing 2% glucose and 100 µg/mL ampicillin. On day 2, the colonies were scraped off the plate and stored in LB with 20% glycerin at −80°C.

### Screening and purification of Nbs

To obtain NP-specific Nbs, three rounds of panning were performed as previously described (47). The ELISA plate was coated with recombinant NP (2 µg/mL) purified from *E. coli* cells and sealed with a 1% PVA solution for 2 h. The original library obtained was added to the ELISA plate and incubated at room temperature for 2 h, after which freshly prepared 0.1 M HCl was added. The mixture was gently shaken at room temperature for 5 min, and 1 M Tris-HCl (pH 8.0) was quickly added to neutralize the mixture. The mixture was added to 1 mL of NEB5αF′ bacteria at the logarithmic stage and cultured at 200 rpm at 37°C for 1 h. M13-assisted phage was added, and the mixture was cultured at 200 rpm at 37°C for 1 h. The mixture was subsequently transferred to 2 × YT/Amp-Kan medium. The first-generation (F1) anti-NP phage library was collected by repeating the above operations. An appropriate amount of eluate was added after the third round of panning. The plate was then diluted, and the mixture was cultured overnight at 37°C. Forty-eight monoclonal antibodies were selected, inoculated into 96-well plates pre-coated with recombinant NP (2 µg/mL), and the supernatants were collected and stored at 4°C. The Nbs that reacted with the NP were screened via indirect ELISA, and all the VHHs of the positive clones were sequenced to obtain the final VHH sequences for analysis. VHH sequences were inserted into the pcDNA3.1 eukaryotic expression vector containing Fc. The plasmid was transfected into HEK293F suspension cells, and the supernatant was collected 7 days later. The supernatant was then filtered through a 0.45 µm filter. Nbs were purified with a Protein A column.

### Enzyme-linked immunosorbent assay

Purified virions (2 µg/mL) or PR8-NP proteins (2 µg/mL) were first immobilized on 96-well microtiter plates. The plates were blocked with 2% BSA for 1 h at room temperature. Gradient-diluted Nbs or Nbs with indicated concentrations were then added and allowed to react at 37°C for 2 h. HRP-conjugated goat anti-human IgG-Fc (diluted 1:8,000) was subsequently added, and the mixture was incubated at 37°C for 0.5 h. Sulfuric acid was sequentially added to the plates to stop the reaction. The data were recorded at 450 nm via an M1000 (Tecan) plate reader.

### Virus infection and titration

Viral infection was performed as previously described (46). All cells were seeded at the desired density in culture plates as per the requirements for different experiments. Viruses were inoculated into cells at a specific multiplicity of infection for different experiments. One hour after inoculation, the medium was replaced with fresh OPTI-MEM containing 0.1 µg/mL of tosylsulfonyl phenylalanyl chloromethyl ketone (TPCK)-trypsin and incubated at 37°C. Virus-containing culture supernatants were collected at the indicated timepoints for titration.

Virus titers of the stocks, cell culture supernatants, and tissue suspensions were determined by end-point titration in MDCK cells or eggs. For end-point viral titration in MDCK cells, each sample was serially diluted with OPTI-MEM containing 0.1 µg/mL of TPCK-trypsin and then inoculated into MDCK cells. Two days after inoculation, supernatants from the inoculated cells were collected and tested for their ability to agglutinate chicken erythrocytes or the expression of GFP, indicating viral replication. The infectious virus titers are reported as log_10_ TCID_50_/mL and were calculated from three replicates using the Reed-Muench method. For end-point viral titration in eggs, 10-fold serial dilutions of each sample were inoculated into 9-day-old SPF eggs. 60 h after inoculation, fluid from the allantoic cavity was collected, and its ability to agglutinate chicken erythrocytes was tested as an indicator of viral replication. The infectious virus titers are reported as log_10_ EID_50_/mL and were calculated from three replicates via the Reed-Muench method (48).

### TAT-Nbs production and purification

The gene fragments for Nb7-TAT or K7.13-TAT were generated by Tsingke Biotechnology Co., Ltd. The pCold II plasmid (Novagen, Darmstadt, Germany), containing a 6-histidine (6 His) tag upstream of the gene insertion site, was used as the expression vector. For Nb expression, the plasmids were transformed into *E. coli* BL21(DE3) after induction with 0.1 mmol/L isopropyl-D-1-thiogalactopyranoside overnight at 16°C. Centrifuged cells were resuspended in lysis buffer (1 × PBS, 0.2 mM PMSF, and 1% Triton X-100) and sonicated for 15 min. After centrifugation, the supernatant was incubated with Ni-BestaRose FF to purify the TAT-Nbs protein, as per the manufacturer’s instructions.

### Co-IP analysis

HEK293T cells or A549 cells were cotransfected with the indicated plasmids with or without virus infection for 24 h. The transfected cells were then harvested and lysed in NP-40 lysis buffer (20 mM Tris-HCl [pH 7.5], 150 mM NaCl, 1% NP-40, 1 mM EDTA with protease inhibitor cocktails). For each immunoprecipitation, 1 mL of lysate or lysate mixture was incubated for 4 h at 4°C with 0.5 µg of the indicated antibody or control IgG and 30 µL of protein A/G-Sepharose (Sigma). The beads were washed three times with 1 mL of lysis buffer containing 500 mM NaCl. The precipitates were then analyzed via standard Western blotting procedures.

### Western blotting

The cells or protein samples were lysed in RIPA buffer (Beyotime, China). Proteins were separated by 10% SDS-PAGE and transferred to a nitrocellulose membrane (Bio-Rad). The membrane was blocked for 1 h in TBST containing 5% milk and subsequently incubated with primary antibodies for 2 h. After a 1-h incubation with an HRP-conjugated secondary antibody, the immunoreactive bands were visualized using an e-BLOT system (e-BLOT Life Science, China). The intensities of the target bands were quantified by using the Image J program (NIH, USA).

### Affinity measurements

Affinity tests were performed on the Biacore T200 instrument. Briefly, the purified NP proteins were coupled and fixed to the M5 chip and activated. Nb7 was diluted from 25 nM to 1.5625 nM via a buffer solution (10 mM Tris-HCl, pH 7.4, 150 mM NaCl, 0.05% Tween 20) and flowed uniformly through the chip (20 µL/min). Finally, competitive elution was performed with a pH 4 glycine solution. At the same time, the negative control was set to remove the nonspecific binding signal, and the kinetic affinity constant was obtained by applying the binding model.

### Atomic model building and refinement

The model of Nb was done using the ColabFold version of AlphaFold 3 (25). Structures were analyzed and figures were generated using Open-Source PyMOL version 2.5.0. (http://www.pymol.org). The ClusPro 2.0 (49) server was used in “antibody mode” to produce docking models between Nb7 and NP or vRNP complex. The top 30 models returned by the server were ranked depending on energy and cluster size. The 10 best ClusPro docking models were further analyzed based on interactions and interface properties calculated according to PDBePISA (Proteins, Interfaces, Structures, and Assemblies, https://www.ebi.ac.uk/pdbe/prot_int/pistart.html) (50). The final model subsequently underwent manual analysis and image generation in Open-Source PyMOL version 2.5.0.

### Indirect IFA

An indirect IFA was performed as previously described (39). The infected or transfected cells were fixed with 4% paraformaldehyde and then washed three times with PBS. The cells were next permeabilized with 0.5% Triton X-100 in PBS and blocked with 5% skim milk for 1 h. Then, the cells were incubated with the indicated primary and secondary antibodies and counterstained with DAPI. The stained cells were observed with a Zeiss microscope (LSM 980). At least 30 cells were randomly selected for quantification in each experiment. The fluorescence intensity was quantified, and colocalization analysis was conducted with ImageJ software.

### Cross-linking immunoprecipitation

At 24 h post-transfection, cells were cross-linked by 1% formaldehyde solution and lysed in cell lysis buffer (10 mM Tris-HCl, pH 7.4, 100 mM NaCl, 0.5% NP-40, 1 mM DTT, 200 units/mL RNaseOUT, and EDTA-free Protease Inhibitor Cocktail). The lysed cell suspension was incubated with anti-NP antibody for 4 h at 4°C. Then, the mixture was incubated with protein A/G magnetic beads and rotated for 4 h at 4°C. The beads were then washed three times with NT2 buffer (50 mM Tris-HCl pH = 7.4, 150 mM NaCl, 1 mM MgCl_2_, and 0.05% NP-40), followed by incubation with 50 µg of proteinase K (Takara Bio) at 55°C for 30 min. Input and co-immunoprecipitated RNAs were recovered by TRIzol extraction and analyzed by qPCR.

### RNA isolation and quantitative PCR

Total RNA was extracted from the cells using TRIzol. In accordance with the manufacturer’s protocol, total RNA was subsequently transcribed into cDNA using M-MLV reverse transcriptase (Promega). The levels of RNA were determined via real-time RT-PCR as described previously (46). Actin was used as an invariant control. Real-time PCR was performed using an ABI 7500 detection system (Applied Biosystems, USA). The RNA level of each gene is shown as a fold change (2^−ΔΔCT^) in the graphs. The sequences of the gene-specific primers used for qPCR are provided in Table S1.

### Dual-luciferase reporter assay

For the viral minigenome assay, HEK293T cells were transfected with pCAGGS constructs expressing the viral PB2, PB1, PA, and NP proteins from the PR8 virus; the pPol I-Luc construct; and an internal control, pRL-TK (Promega), along with other plasmids. The cells were incubated at 37°C for 24 h, and the cell lysates were subsequently prepared via the Dual-Luciferase Reporter Assay System (Promega).

### Subcellular fractionation

The cells were harvested by scraping into cell lysis buffer containing 10 mM HEPES (pH 7.4), 10 mM NaCl, 1 mM KH_2_PO_4_, 5 mM NaHCO_3_, 1 mM CaCl_2_, 0.5 mM MgCl_2_, and 5 mM EDTA, along with a complete protease inhibitor cocktail. The cell lysates were allowed to swell for 5 min, followed by homogenization for 50 strokes. Then, the cells were centrifuged at 800 × *g* for 5 min, generating a pellet containing nuclei and debris and a supernatant consisting of the cytosol and plasma. The pellets were resuspended in 1 mL of TSE buffer containing 10 mM Tris (pH 7.5), 300 mM sucrose, 1 mM EDTA, and 0.1% NP-40, along with a complete protease inhibitor cocktail. The suspension was then pelleted, resuspended, and washed twice with PBS. The final pellets were pure nuclei. The subcellular fractions were subsequently analyzed via Western blotting.

### Mouse study

The prophylaxis and therapy experiments in the mouse model were conducted as described previously (51). For prophylactic assessment, groups of 11 mice were intranasally (i.n.) administered a low (10 mg/kg) or high (30 mg/kg) dose of Nb7 at 12 h before challenge with 10^4^ EID_50_ of the PR8 virus. For therapy, groups of 11 mice were intranasally (i.n.) administered low (10 mg/kg) or high (30 mg/kg) doses at 2 and 24 h after challenge. The control mice were inoculated with IgG and infected in the same way. After inoculation, five mice in each group were monitored daily for 14 days for weight loss and mortality. The other six mice per group were euthanized on days 3 and 5 post-infection, and their lung and nasal turbinate tissues were collected for viral titration or pathological assessments by Servicebio (Servicebio Technology, China).

### Statistical analysis

The data are expressed as the means ± standard deviations. Statistical significance was determined via Student’s two-tailed unpaired *t* test or analysis of variance (ANOVA) with GraphPad Prism software (version 9.0, San Diego, CA, USA). Differences between groups were considered significant when the *P*-value was <0.05 (*), <0.01 (**), <0.001 (***), or <0.0001 (****). “ns” indicates no significant difference.

## Data Availability

All data are included in the article and Supplementary data.

## SUPPLEMENTAL MATERIAL

Supplemental material jvi.01478-25-s0001.pdf Figures S1 to S4; Table S1.

ASM does not own the copyrights to Supplemental Material that may be linked to, or accessed through, an article. The authors have granted ASM a non-exclusive, world-wide license to publish the Supplemental Material files. Please contact the corresponding author directly for reuse.
