# Supplementary material for: Broad-spectrum inhibition of influenza A virus replication by blocking the nuclear export of viral ribonucleoprotein complexes
Source: J Virol. 2025 Nov 25;99(12):e01478-25. doi: 10.1128/jvi.01478-25 (PMC12724358; doi:10.1128/jvi.01478-25)
Supplement: Supplemental material — Figures S1 to S4; Table S1. [file jvi.01478-25-s0001.pdf]

Fig. S1

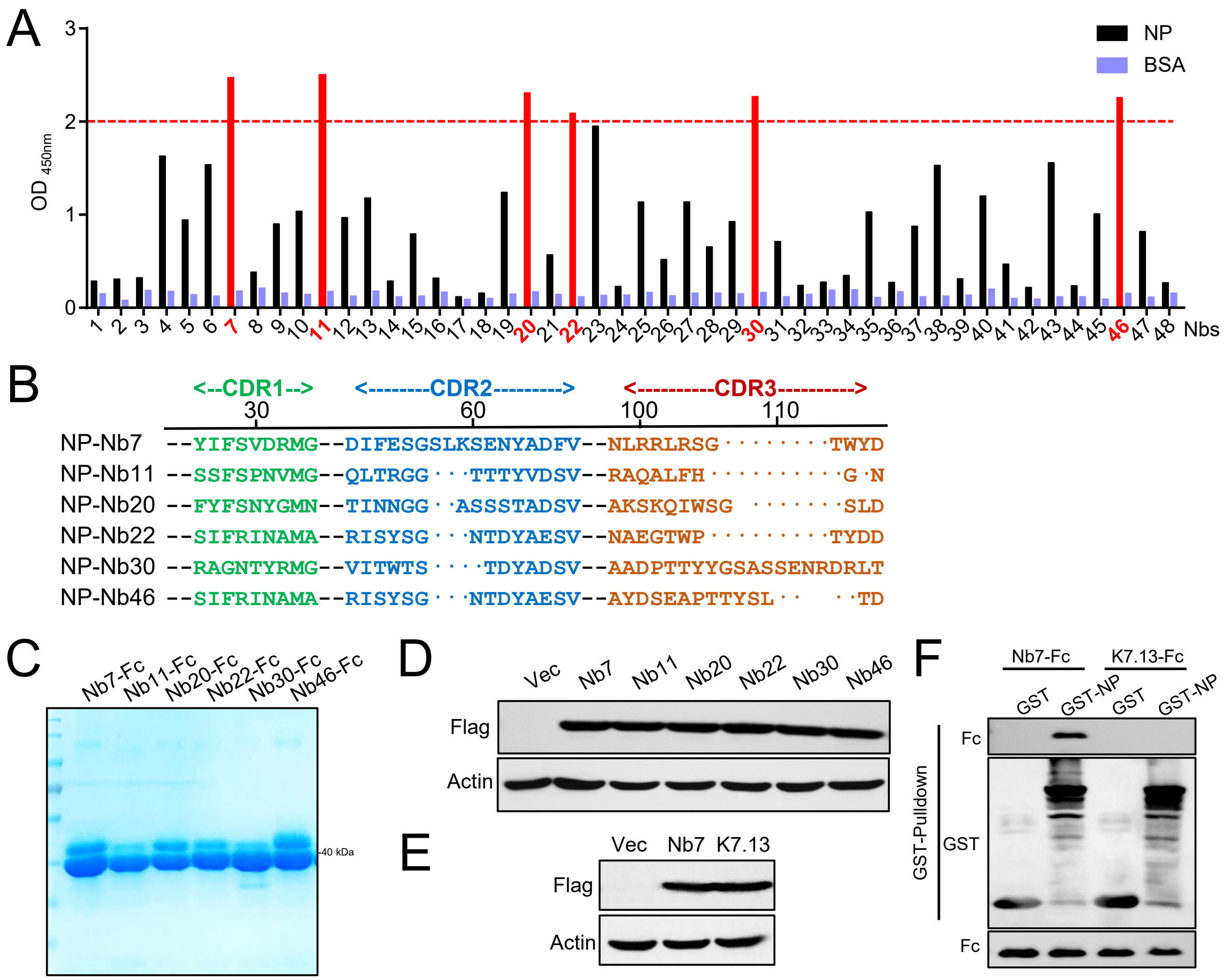


**Fig S1. Generation and characterization of NP-specific Nbs.** (A) Phage-ELISA to screen the Nbs highly reacted with NP. Nbs with an absorbance value greater than 2 are marked in red. (B) Amino acid sequence comparison of six strains of anti-NP Nbs. The CDR areas are distinguished by different colors. (C) The Nbs-Fc were purified and analyzed by SDS-PAGE. (D) A549 cells were transfected with the indicated Nbs expression plasmid or empty vector (Vec). After 24 h, the cells were infected with the PR8-Nluc virus for an additional 24 h, and the cell lysates were subjected to Western blotting analysis. (E) A549 cells were transfected with Nb7-Flag, K7.13-Flag, or Vec for 24 h before infection with the PR8 virus (H1N1, MOI = 0.01). The cell lysates were subjected to Western blotting analysis. (F) Nb7 interacts with NP directly. Purified GST-NP was used to pull down purified Nb7-Fc. The immunoprecipitants were analyzed by Western blotting.

Fig. S2

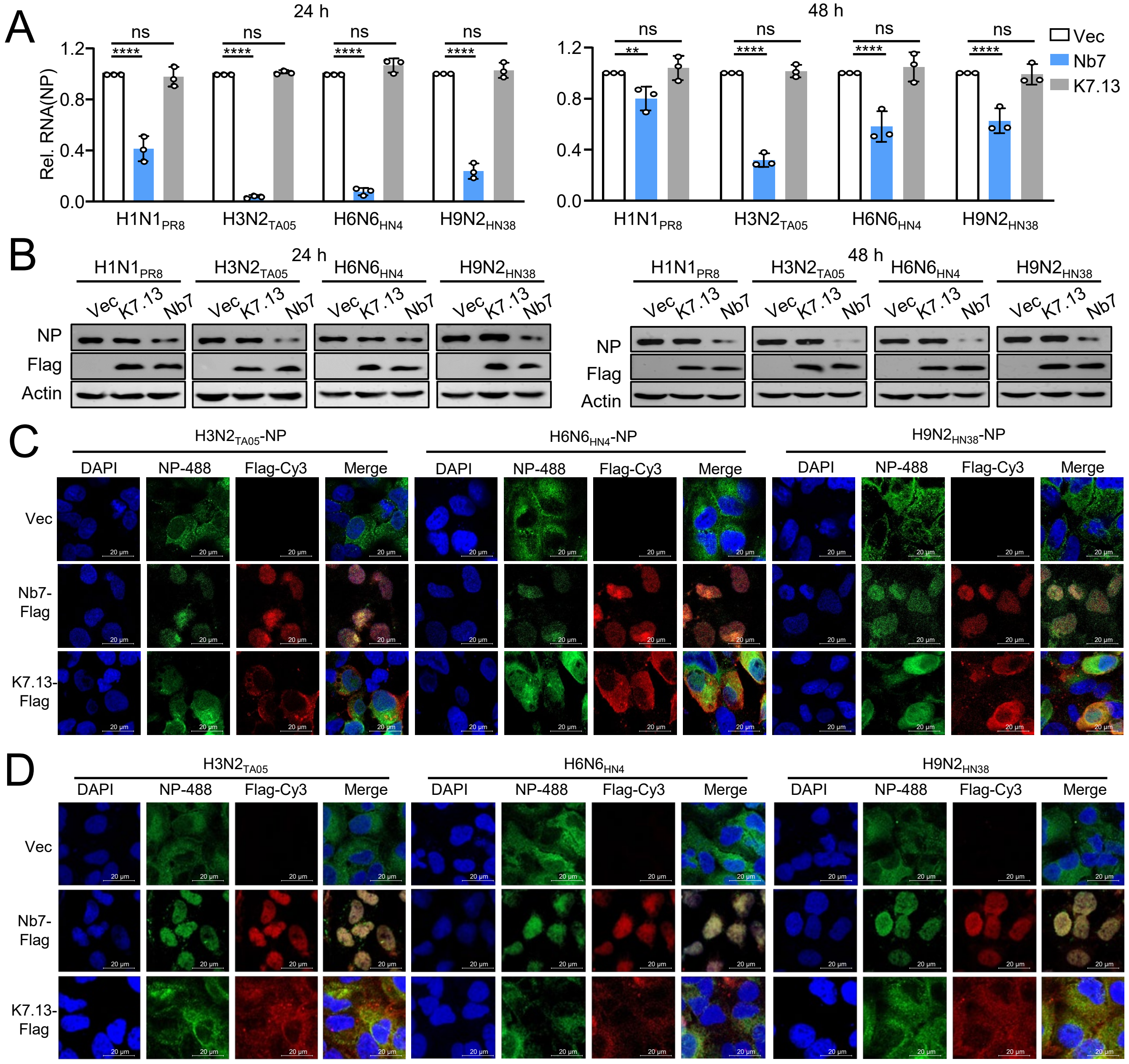


**Fig S2. Nanobody Nb7 inhibits replication of different subtypes of IAVs.** (A-B) A549 cells were transfected with Nb7-Flag, K7.13-Flag, or Vec. After 24 h, the cells were infected with PR8 (H1N1), TA05 (H3N2), HN4 (H6N6) or HN38 (H9N2) viruses (MOI=0.1). At 24 h and 48 h post-infection, total RNA was extracted for qPCR to detect the expression of viral RNA (A), and the cell lysates were collected for Western blotting (B). (C) A549 cells were transfected with Nb7-Flag, K7.13-Flag, or Vec, along with $H1N1_{PR8}$-NP, $H3N2_{TA05}$-NP, $H6N6_{HN4}$-NP or $H9N2_{HN38}$-NP. After 18 h, the cells were fixed and stained with anti-Flag and a commercial anti-NP mAb. (D) A549 cells were transfected with Nb7-Flag, K7.13-Flag, or Vec. After 24 h, the cells were infected with TA05 (H3N2), HN4 (H6N6) or HN38 (H9N2) viruses (MOI=0.1) for 24 h and stained with anti-Flag (diluted 1:300) and a commercial anti-NP mAb (diluted 1:300). The data shown represent three independent experiments (n = 3); bars represent the mean ± SD.

Fig. S3

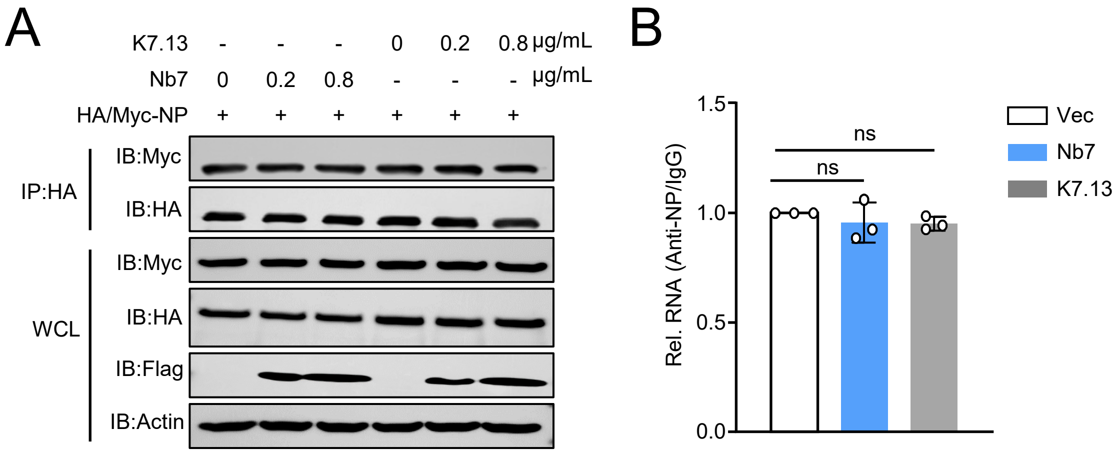


**Fig S3. Nb7 does not affect the oligomerization and vRNA binding of NP.** (A) HEK293T cells were transfected with plasmids encoding Myc/HA-NP and different doses of Nbs for 24 h before being subjected to co-IP with anti-HA antibody. (B) HEK293T cells were transfected with the Nb7-Flag, K7.13-Flag, or Vec, along with the PB2, PB1, PA, NP, and pPol I-vRNA. After 24 h, the cells were subjected to the cross-linking RNA immunoprecipitation assay with a commercial anti-NP mAb or IgG. The level of vRNA was quantified by qPCR and normalized to IgG. The data shown represent three independent experiments (n = 3); bars represent the mean ± SD.

Fig. S4

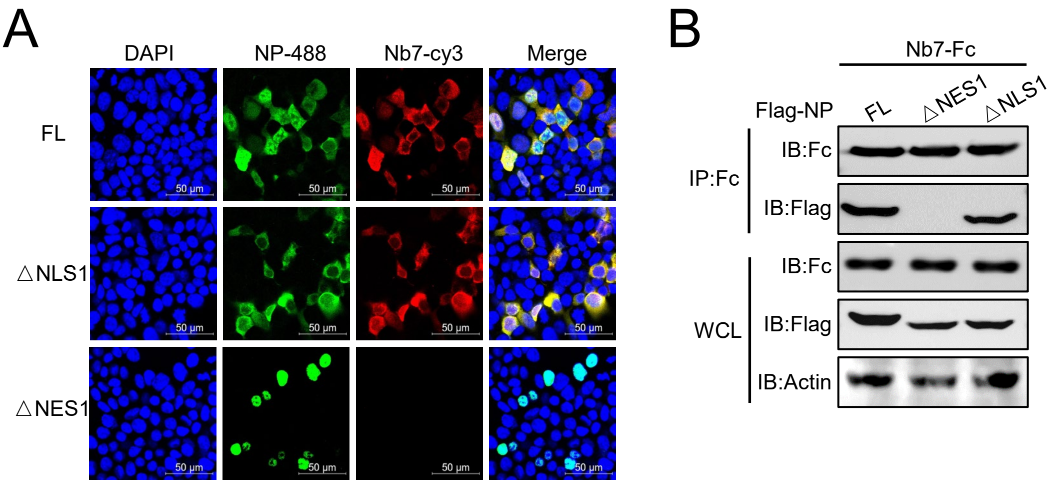


**Fig S4. NES1 is the key for Nb7 to recognize NP.** (A) A549 cells were transfected with plasmids expressing full-length (FL) NP, NP-ΔNLS1, or NP-ΔNES1. After 18 h, the cells were fixed and stained with commercial anti-NP mAb and Nb7-Fc. (B) HEK293T cells were transfected with plasmids expressing NP-FL, NP-ΔNLS1, or NP-ΔNES1. After 24 h, cell lysates were co-immunoprecipitated with anti-human Fc.

**Table S1**. Primers used in this study.

| Name | Sequence (5' - 3') | Application |
|---|---|---|
| Nb-His-F | ATACACCATGAATCACAAAGCTTCTTCAGGTGCAGCTCGT | Construction |
| Nb-His-R | GTCGTCATCCTTGTAATCTCTAGATGAGGAGACGGTG | |
| Nb-Fc-F | TCTTGCACTTGTCACGAATTCATGCAGGTGCAGCTCGTGGAG | Construction |
| Nb-Fc-R | TTTGTCACAAGATTTGGGCTCTTTACCCGGAGACAGGGA | |
| Nb-Flag-F | CATTTTGGCAAA GAATTCATGCAGGTGCAGCTCGTGGAG | Construction |
| Nb-Flag-R | GTCGTCATCCTTGTAATCTGAGCTGACGGTGACCTGGG | |
| TAT-Nb-F | ATACACCATGAATCACAAAGCTTCTTACGGTCGTAAGAA | Construction |
| TAT-Nb-R | GTCGTCATCCTTGTAATCTCTAGATGAGCTGACGGTGAC | |
| $NP^{Q42A}$-F | ACGATTCTACATCGCAATGTGCACCGAACTTAAACTCAG | Mutation |
| $NP^{Q42A}$-R | CTGAGTTTAAGTTCGGTGCACATTGCGATGTAGAATCGT | |
| $NP^{E46A}$-F | TACATCCAAATGTGCACCGCACTTAAACTCAGTG | Mutation |
| $NP^{E46A}$-R | CACTGAGTTTAAGTGCGGTGCACATTTGGATGTA | |
| $NP^{K48A}$-F | TCCAAATGTGCACCGAACTTGCACTCAGTGATTATGAGG | Mutation |
| $NP^{K48A}$-R | CCTCATAATCACTGAGTGCAAGTTCGGTGCACATTTGGA | |
| $NP^{Q42A+E46A}$-F | ACGATTCTACATCGCAATGTGCACCGCACTTAAACTCAGTG | Mutation |
| $NP^{Q42A+E46A}$-R | CACTGAGTTTAAGTGCGGTGCACATTGCGATGTAGAATCGT | |
| $NP^{Q42A+K48A}$-F | GATTCTACATCGCAATGTGCACCGAACTTGCACTCAGTGATTAT | Mutation |
| $NP^{Q42A+K48A}$-R | ATAATCACTGAGTGCAAGTTCGGTGCACATTGCGATGTAGAATC | |
| $NP^{E46A+K48A}$-F | TCTACATCCAAATGTGCACCGCACTTGCACTCAGTGATTATGAG | Mutation |
| $NP^{E46A+K48A}$-R | CTCATAATCACTGAGTGCAAGTGCGGTGCACATTTGGATGTAGA | |
| $NP^{Q42A+E46A+K48A}$-F | CGATTCTACATCGCAATGTGCACCGCACTTGCACTCAGTGATTA | Mutation |
| $NP^{Q42A+E46A+K48A}$-R | AATCACTGAGTGCAAGTGCGGTGCACATTGCGATGTAGAATCG | |
| β-actin-F | GTCGTCGACAACGGCTCCGGCATG | qRT-PCR |
| β-actin-R | ATTGTAGAAGGTGTGGTGCCAGAT | |
| SeV-NP-F | CAAGAGCCCACTCTTCCAGGG | qRT-PCR |
| SeV-NP-R | CTGAACGCCTCTAACCTGTTG | |
| VSV-NP-F | ACGGCGTACTTCCAGATGG | qRT-PCR |
| VSV-NP-R | CTCGGTTCAAGATCCAGGT | |
| NDV-NP-F | CAGTATTCACCCTAAACAGTG | qRT-PCR |
| NDV-NP-R | CCTCGCTAACAGCAATCC | |
| IAV-NP-F | GCGCCAAGCTAATAATGGTG | qRT-PCR |
| IAV-NP-R | GGAGTGCCAGATCATCATGT | |
